# Hippocampal sulcal cavities: prevalence, risk factors and association with cognitive performance. The SMART-Medea study and PREDICT-MR study

**DOI:** 10.1007/s11682-018-9916-y

**Published:** 2018-07-06

**Authors:** Kim Blom, Huiberdina L. Koek, Yolanda van der Graaf, Maarten H. T. Zwartbol, Laura E. M. Wisse, Jeroen Hendrikse, Geert Jan Biessels, Mirjam I. Geerlings, on behalf of the SMART Study Group

**Affiliations:** 1 Julius Center for Health Sciences and Primary Care, University Medical Center Utrecht, Stratenum 6.131, P.O. Box 85500, 3508 GA Utrecht, the Netherlands; 2 Department of Geriatrics, University Medical Center Utrecht, Utrecht, the Netherlands; 3 Department of Radiology, University Medical Center Utrecht, Utrecht, the Netherlands; 4 Penn Image Computing and Science Laboratory, Department of Radiology, University of Pennsylvania, Philadelphia, PA USA; 5 Department of Neurology, University Medical Center Utrecht, Utrecht, the Netherlands

**Keywords:** Hippocampus, (dilated) perivascular spaces, Cognition, Magnetic resonance imaging

## Abstract

Hippocampal sulcal cavities (HSCs) are frequently observed on MRI, but their etiology and relevance is unclear. HSCs may be anatomical variations, or result from pathology. We assessed the presence of HSCs, and their cross-sectional association with demographics, vascular risk factors and cognitive functioning in two study samples. Within a random sample of 92 patients with vascular disease from the SMART-Medea study (mean age = 62, SD = 9 years) and 83 primary care patients from the PREDICT-MR study (mean age = 62, SD = 12 years) one rater manually scored HSCs at 1.5 T 3D T1-weighted coronal images blind to patient information. We estimated relative risks of age, sex and vascular risk factors with presence of HSCs using Poisson regression with log-link function and robust standard errors adjusted for age and sex. Using ANCOVA adjusted for age, sex, and education we estimated the association of the number of HSCs with memory, executive functioning, speed, and working memory. In the SMART-Medea study HSCs were present in 65% and in 52% in the PREDICT-MR study (χ^2^ = 2.99, df = 1, *p* = 0.08). In both samples, no significant associations were observed between presence of HSCs and age (SMART-Medea: RR = 1.00; 95%CI 0.98–1.01; PREDICT-MR: RR = 1.01; 95%CI 0.99–1.03), sex, or vascular risk factors. Also, no associations between HSCs and cognitive functioning were found in either sample. HSCs are frequently observed on 1.5 T MRI. Our findings suggest that, in patients with a history of vascular disease and primary care attendees, HSCs are part of normal anatomic variation of the human hippocampus rather than markers of pathology.

**Electronic supplementary material:**

The online version of this article (10.1007/s11682-018-9916-y) contains supplementary material, which is available to authorized users.

## Introduction

The hippocampus plays an important role in memory and cognitive processes (Squire 1992). Volume reduction of the hippocampus is a common finding on brain magnetic resonance imaging (MRI) and is part of normal aging as well as of Alzheimer’s disease (AD) (Small et al. 2011). Apart from volume reduction, hippocampal sulcal cavities (HSCs) are frequently observed on MRI, which appear as small changes in signal intensity, isointense to cerebrospinal fluid (CSF). It has been estimated that HSCs are observed as frequent as 47% in healthy persons and in 66% of patients with impaired memory (Maller et al. 2011). Also, the prevalence of HSCs may be positively correlated with age (Maller et al. 2011).

The etiology and clinical relevance of HSCs is not well understood. Several studies observed that HSCs are normal variations originating from embryonal folding of the hippocampus (Kier et al. 1997; Hayman et al. 1998). According to others, HSCs are dilated perivascular spaces (dPVS), also known as dilated Virchow-Robin spaces (dVRS) (Kwee and Kwee 2007). Previous studies found a higher number of HSCs with an increasing mean arterial pressure (MAP) (Van Veluw et al. 2013), and with higher systolic and diastolic blood pressure (Yao et al. 2014), suggesting that the etiology of HSCs may be related to vascular factors*.* However, a review reported that the relation between HSCs and hypertension has been questioned and that some studies excluded patients with hypertension (Maller et al. 2011). Another hypothesis is that HSCs are due to an underlying ischemic process, consistent with studies that found that small cavities in the hippocampus were small infarcts (Donnani and Norrving 2009). Recently, when we used 7 T MRI and histopathology, we found indications that some HSCs may be of an ischemic subtype, while other HSCs are filled with CSF and are not presumed to be due to ischemia (Van Veluw et al. 2013).

Clinical relevance of the presence of HSCs is unclear. The number of HSCs may influence cognitive functioning in people with cognitive complaints, as one study that included subjects with subjective progressive memory loss found an effect on a speed-task (Bartrés-Faz 2001). However, other studies with participants from the community did not find an association with cognitive functioning (Maclullich et al. 2004; Yao et al. 2014).

To further unravel the etiology and clinical relevance of HSCs, we aimed to 1) estimate and compare the occurrence of HSCs in patients with a history of vascular disease from the SMART-Medea study and in primary care attendees from the PREDICT-MR study; 2) estimate the cross-sectional associations of age, sex, and vascular risk factors with presence of HSCs; and 3) estimate the cross-sectional association of HSCs with cognitive functioning.

## Methods

### Study populations

#### SMART-Medea study

The Second Manifestations of ARTerial disease-Memory, depression and aging (SMART-Medea) study is an ongoing prospective cohort study aimed to investigate brain changes on MRI, late-life depression and cognitive decline in patients with a history of vascular disease (Grool et al. 2011). The SMART-Medea study started in 2006 as an ancillary study to the SMART-MR study, of which rationale and design have been described previously (Geerlings et al. 2009; Geerlings et al. 2010). In brief, from 2001 to 2005, 1309 middle-aged and older adult patients with coronary artery disease, cerebrovascular disease, peripheral arterial disease, or an aneurysm of the abdominal aorta were included in the SMART-MR study*.* Between January 2006 and May 2009, 754 participants had follow-up measurements for the SMART-MR cohort. From April 2006, measurements were added as part of the SMART-Medea study including depression assessment, psychosocial risk factor questionnaires, saliva sampling for stress hormones, and a 3-dimensional T1-weighted MR image to assess hippocampal volumes. During a one day visit to the University Medical Center Utrecht participants underwent a physical examination, ultrasonography of the carotid arteries, sampling of blood and urine, neuropsychological and depression assessment, and a 1.5 T brain MRI scan. Questionnaires were used for assessing demographics, risk factors and medical history, medication use, functioning, psychosocial vulnerability and stress factors, and depressive symptoms.

The SMART-MR and SMART-Medea studies were approved by the ethics committee and written informed consent was obtained from all participants.

Of the 710 patients that were included in the SMART-Medea study, 636 had volumetric measurements of the hippocampus at MRI. For the present study, 100 patients were randomly selected (Knoops et al. 2009), of whom 92 had HSCs data available. Four patients were excluded because of dementia, one because of missing mini-mental state examination score, one due to movement artefacts in the MR images, and two because of a difference in signal intensity of the MR images.

### PREDICT-MR study

The PREDICT-Magnetic Resonance (PREDICT-MR) study aims to investigate risk factors and brain changes on MRI in primary care attendees not selected on complaints or disease (Wisse et al. 2012). The PREDICT-MR study is an ancillary study to the PREDICT-NL study, the Dutch part of the Predict Depression (PredictD) study, which is a multicenter international prospective cohort study to predict risk of onset of major depressive disorder in primary care patients (Stegenga et al. 2012a; King et al. 2008). Detailed study designs of PredictD and PREDICT-NL have been described elsewhere (King et al. 2008; Stegenga et al. 2012a, b). For these studies, in 2003 consecutive adult patients (aged ≥18 years) who were in the waiting room of their general practitioner were asked to participate, irrespective of their reason for consulting their general practitioner. Between June 2010 and January 2012, participants of the PREDICT-NL study were invited to participate in the PREDICT-MR study. People were eligible for participation in the PREDICT-MR study when they were not severely ill or did not have a diagnosis of dementia. Participants underwent brain MRI, a diagnostic depression interview, blood sampling, questionnaires, neuropsychological testing and a clinical assessment at the UMC Utrecht.

The PREDICT-MR and PREDICT-NL studies were approved by the medical ethics committee and written informed consent was obtained from all participants.

Of the 125 patients that were included in the PREDICT-MR study, 84 had adequate volumetric measurements of the hippocampus at MRI available and were included in the analysis: 29 participants had no MRI; six had no T1 sequence; and in six participants the resolution was too low for hippocampus segmentation.

### Risk factors and covariates

For both study samples, risk factors were measured in a similar way during the visit to the medical center. Educational level was obtained in 8 categories according to the Dutch educational system and then grouped into 3 levels for baseline characteristics. Low level of education included no education or primary school only (comparable to up to 6 years of education), high level education included higher professional education and (pre-)university education (comparable to ≥15 years of education), and all other educational levels were defined as an intermediate level of education (comparable to around 7–14 years of education). In the analyses, we entered the variable with 8 categories.

Blood pressure was measured three times in supine position and the average was calculated. Hypertension was defined as mean systolic blood pressure ≥ 140 mmHg, mean diastolic blood pressure ≥ 90 mmHg or use of antihypertensive drugs. Hyperlipidemia was defined as use of lipid-lowering drugs, or a cholesterol ratio ≥ 5.0 which was calculated by using fasting levels of cholesterol and the formula: total cholesterol/high density lipoprotein cholesterol. Overweight was defined as a body mass index (BMI) ≥25.0 by measuring height and weight without shoes or heavy clothing calculated as weight (kg)/height (m)^2^. Diabetes mellitus (DM) was defined as use of glucose-lowering agents or insulin, a known history of DM, non-fasting plasma glucose ≥11.1 mmol/L, or fasting plasma glucose ≥7.0 mmol/L. Pack years of smoking were calculated by use of a questionnaire on smoking habits.

### MRI protocol: SMART-Medea study

The MR images were obtained using a 1.5Twhole-body system (Gyroscan ACS-NT, Philips Medical Systems, Best, the Netherlands). The protocol consisted of a transversal T1-weighted gradient-echo sequence (repetition time (TR)/echo time (TE): 235/2 ms; flip angle, 80°), a transversal T2-weighted turbo spin-echo sequence (TR/TE:2200/11 ms and 2200/100 ms; turbo factor 12), a transversal T2-weighted fluid attenuating inverse recovery (FLAIR) sequence (TR/TE/inversion time (TI): 6000/100/2000 ms) and a transversal inversion recovery (IR) sequence (TR/TE/TI: 2900/22/410 ms) (field of view (FOV) 230 × 230 mm; matrix size, 180 × 256; slice thickness, 4.0 mm; no gap; 38 slices) (Geerlings et al. 2009; Knoops et al. 2010). For hippocampus volumes T1-weighted 3D fast-field-echo(FFE) sequences were performed (TR/TE: 7.0/3.2 ms; flip angle, 8°; FOV 240 mm; matrix size, 240 × 256; slice thickness 1.0 mm; no gap; 170 slices) (Knoops et al. 2009).

### MRI protocol: PREDICT-MR study

The MR images were obtained using a 1.5 T MRI whole-body system (Gyroscan ACS-NT, Philips Medical Systems, Best, The Netherlands). For outlining of intracranial volume (ICV) the protocol consisted of a high resolution 4.00 × 0.90 × 1.03 mm T2-weighted turbo spin-echo sequence (TSE) (TR/TE: 2200/11/100 ms; flip angle, 90°; turbo factor 12; matrix size, 38x256x168). For hippocampus volumes high resolution 1.10 × 1.10 × 1.10 mm T1-weighted 3D FFE sequences were performed (TR/TE: 7.0/3.2 ms; flip angle 8°; matrix size, 240x240x240).

### Brain segmentation: SMART-Medea study

The T1-weighted gradient-echo, IR sequence, and FLAIR sequence were used for brain segmentation according to the k-nearest neighbor (KNN) classification, as has been described elsewhere (Anbeek et al. 2004, 2005). It distinguishes gray matter, white matter, sulcal and ventricular cerebrospinal fluid (CSF), and lesions. The results of the segmentation analysis were visually checked for the presence of infarcts and adapted if necessary, to make a distinction between white matter lesions and infarct volumes. Total brain volume was calculated by summing the volumes of gray and white matter and, if present, the volumes of WMLs and infarcts. All volumes cranial to the foramen magnum were included. As a result, the total brain volume included the cerebrum, brainstem, and cerebellum. Total intracranial volume (ICV) was calculated by summing up total brain and CSF volume.

Hippocampal volumes were assessed and described in detail before (Knoops et al. 2012). Briefly, the sagittal T1-weighted images were tilted to the coronal plane and orientated perpendicular to the long axis of the left hippocampus. The hippocampus was manually outlined by two trained investigators, blinded to all clinical information, on an average of 40 slices and included the hippocampus proper, subiculum, fimbria, alveus, and dentate gyrus.

### Brain segmentation: PREDICT-MR study

All segmentation were performed using in-house developed software based on MeVisLab (MeVis Medical Solutions AG, Bremen, Germany) (Ritter et al. 2011). Hippocampus segmentations included subiculum, cornu ammonis sectors, the dentate gyrus and alveus and fimbria. HSCs were excluded from the hippocampus mask. Hippocampal volumes were segmented by one rater (LEMW) in the coronal plane according to the previously published protocol for 7 T (Wisse et al. 2012, 2014), with two adjustments. The medial border, located at the most medial part of the grey matter of the temporal stem, was kept consistent in the transition from hippocampal body to tail to obtain a smoother segmentation. This border was kept until the last slices where the hippocampal grey is mainly surrounded by the splenium and is separated from its medial aspect which is not included in the segmentation. Additionally, because the alveus and fimbria are difficult to separate from the hippocampal grey matter at 1.5 T, they were included in the segmentation following the EADC-ADNI Harmonized protocol for hippocampus segmentation, except for the first one or two slices where the alveus was difficult to discern on the coronal slices (Boccardi et al. 2015). The ICC for the total hippocampal formation for 10 randomly chosen was 0.86. Two raters (LEMW and PHV), blinded to subject information, manually segmented ICV on transversal slices. The first and the last slice were segmented along with every other slice in-between. The other slices were interpolated using MeVisLab. All interpolated ICV segmentations were inspected visually. The ICC for ICV for LEMW for repeated tracing in 6 randomly chosen subjects was 0.99. The ICC for ICV PHV and LEMW in 10 randomly chosen subjects was 0.90.

### Segmentation of hippocampal sulcal cavities

For both studies HSCs were manually segmented in the coronal plane by one rater (MHTZ), a neuro-radiologist in training with over five years of experience. The rater was blind to participant information with exception of study sample status, using in-house developed software (Kuijf 2013) based on MevisLab (Ritter et al. 2011). HSCs were defined by the following properties: 1) hypointense signal on T1-weighted images, 2) located in regions adjacent to where we would expect the stratum radiatum lacunosum moleculare, 3) not connected with the temporal horn of the lateral ventricle, which, in case of doubt, was checked in the sagittal plane, and 4) preferably present on multiple consecutive slices. We did not measure size or rate small or large size when scoring HSCs. The minimum size is expected to be around 1 mm because HSCs can be visually detected from about one voxel size on the 3D T1 images we used, which was 1 mm^3^. Smaller HSCs cannot be detected due to the partial volume effect, which makes the HSCs undiscernible from the surrounding tissue. The intra-rater reliability (intraclass correlation coefficient), which was estimated in a random sample of 10 participants from both samples (in total *n* = 20) was 0.83 for the SMART-Medea sample and 0.96 for the PREDICT-MR sample.

### Neuropsychological examination

Cognitive functioning was assessed with a set of standard neuropsychological tests. Memory functioning was assessed with the 15 Word Learning Test (15-WLT) (immediate recall based on 5 trials and delayed recall) (Brand and Jolles 1985) and the delayed recall of the Rey-Osterrieth Complex Figure test (Osterrieth 1944). Working memory was assessed with the longest span scores Forward Digit Span and Backward Digit Span (Wechsler 2008). Executive functioning was assessed by the Visual Elevator test (Robertson et al. 1996) (10 trials), the Brixton Spatial Anticipation test (Burgess and Shallice 1996) and Verbal Fluency tests (letter ‘A’ with a time span of 60 s and category ‘animal’ with a time span of 120 s) (Wilkins et al. 1987). Speed was assessed by the Digital Symbol Substitution Test (Lezak et al. 2004) (120 s). Mean scores of both study samples are provided in supplemental table 1.

Composite *z* scores were calculated for each study sample separately, for four domains: memory (MEM), working memory (WMEM), executive functioning (EXEC), and information processing speed (SPEED). MEM included the immediate and delayed recall of the 15-WLT, and the delayed recall of the Rey-Osterrieth Complex Figure test; WMEM included the longest span scores of the Forward and Backward Digit Span; EXEC included the Visual Elevator test, Brixton Anticipation test and the Verbal Fluency tests; SPEED was a direct derivative from the *z* score of the Digital Symbol Substitution Test and did not include other tests. The Visual Elevator test scores were transformed with a natural logarithm before they were multiplied by minus 1, so that higher scores represented better performance. The Brixton Spatial Anticipation test scores were also multiplied by minus 1 so that higher scores represented better performance. Composite scores were computed by converting all raw scores ((individual test score - mean test score) / standard deviation) to *z* scores and averaging these for each domain before the final *z* transformation.

### Data analysis

First, baseline characteristics were calculated for the SMART-Medea sample (*n* = 92) and for the PREDICT-MR sample (*n* = 84). Next, using Chi-square tests, we tested if the presence (yes/no HSCs) or number (three groups: 0, 1 or 2 or more HSCs present) of HSCs differed between both study samples. All further analyses were performed for both samples separately. First, using ANCOVA we explored if total hippocampal volume and ICV differed according to HSCs groups, adjusting for age, sex and education when ICV was the dependent variable, and additionally for ICV when hippocampal volume was the dependent variable. Assumptions of homogeneity for the ANCOVA were tested initially with a Levene’s test of equality of error variances; there were no violations of these homogeneity assumptions. Second, using Poisson regression models with log-link function and robust standard errors we estimated relative risks (RR) of age, sex, education and vascular risk factors (cerebrovascular disease, hypertension, overweight, hyperlipidemia, diabetes mellitus and smoking in pack years) with presence or absence of hippocampal cavities. Relative risks (RR) instead of odds ratio’s (OR) are recommended to prevent overestimation of the true risk when an outcome is common (>10%). (Knol et al. 2012). A Poisson regression analysis with log-link function and robust standard errors provides RR and therefore we used these analyses in this study, as cavities were common. In the first model, no adjustments were made, and in the second model we adjusted for age and sex. Last, using linear regression analyses, we estimated cross-sectional associations between categories of numbers (0, 1, 2 or more) of HSCs as the independent variable, and the continuous z-scores of the different cognitive domains as the dependent variable. Analyses were performed unadjusted and adjusted for age, sex and educational level.

Statistical analyses were performed using IBM SPSS Statistics for Windows, Version 21.0.

## Results

Baseline characteristics of both study populations are displayed in Table 1. Participants were of similar age (mean 62 years). In the SMART-Medea sample, 21% were female, 71% had hypertension, and 28% had a history of cerebrovascular disease. In the PREDICT-MR study 56% were female, 54% had hypertension, and none had a history of cerebrovascular disease.

**Table 1:** Baseline characteristics of the study samples

Characteristics		SMART-Medea	PREDICT-MR
		n = 92	n = 84
Age (years)	Mean age of all patients ± S.D.	62.2 ± 9.4	61.5 ± 11.8
Sex	Male	73 (79.3%)	37 (44.0%)
	Female	19 (20.7%)	47 (56.0%)
Education	No education or primary school only	9 (9.8%)	2 (2.4%)
	Intermediate level education	55 (59.8%)	57 (67.9%)
	High level education	28 (30.4%)	25 (29.7%)
Cerebrovascular disease	No	66 (71.7%)	83 (100%)
	Yes	26 (28.3%)	0 (0.0%)
Hypertension	No	27 (29.3%)	39 (46.4%)
	Yes	65 (70.7%)	45 (53.6%)
Hyperlipidaemia	No	23 (25.8%)	60 (75.9%)
	Yes	66 (74.2%)	19 (24.1%)
Overweight	No	24 (26.1%)	36 (42.9%)
	Yes	68 (73.9%)	48 (57.1%)
Diabetes Mellitus	No	71 (77.2%)	69 (85.2%)
	Yes	21 (22.8%)	12 (14.8%)
Smoking	Never	9 (9.8%)	35 (41.7%)
	Former	58 (63.0%)	37 (44.0%)
	Current	25 (27.2%)	12 (14.3%)
	Pack years, mean ± S.D.	26.0 ± 21.7	11.8 ± 18.8
Presence of HSC	No HSC	32 (34.8%)	40 (47.6%)
	1 HSC	22 (23.9%)	20 (23.8%)
	2 or more HSC	38 (41.3%)	24 (28.6%)
Number of hippocampal cavities (n) median (range).		1 (0–8)	1 (0–7)
Total hippocampal volume (mL), mean ± S.D.		5.9 ± 0.7	6.6 ± 0.8
Intracranial volume (mL), mean ± S.D.		1442.8 ± 124.1	1430.3 ± 153.0

Within the SMART-Medea study 65% (median 1; range 0–8) of the patients had HSCs and within the PREDICT-MR study 52% (1; 0–7) participants had HSCs (Table 1) (χ^2^ = 2.99, df = 1, *p* = 0.08). Categories of HSCs numbers (0, 1 or 2 or more) did not differ significantly between the cohorts (χ^2^ = 3.79, df = 1, *p* = 0.15).

There was no significant difference in intracranial volume between subjects with 0, 1, and 2 or more HSCs in either study sample (Table 2). Also, hippocampal volume did not differ in the SMART-Medea sample, but in the PREDICT-MR sample hippocampal volume was significantly larger in participants with ≥2 HSCs compared to those with no HSCs (adjusted mean difference 0.47 mL, 95% CI: 0.13; 0.82, *p* < 0.01).

**Table 2:** Adjusted mean hippocampal volume (mL) and intracranial volume (ICV) (mL) according to number of HSC in the SMART-Medea and PREDICT-MR cohort

	SMART-Medea			
	Mean hippocampal volume^a^	Mean difference in hippocampal volume (95% CI)	Mean ICV^b^	Mean difference in ICV (95% CI)
No HSCs (n = 32)	5.98	-- (reference)	1433.2	-- (reference)
1 HSCs (*n* = 22)	5.89	−0.09 (−0.44; 0.27)	1453.6	20.5 (−36.8; 77.7)
≥ 2 HSCs (*n* = 38)	5.93	−0.05 (−0.36; 0.26)	1444.6	11.4 (−38.5; 61.3)
	PREDICT-MR			
	Mean hippocampal volume^a^	Mean difference in hippocampal volume (95% CI)	Mean ICV^b^	Mean difference in ICV (95% CI)
No HSCs (n = 40)^c^	6.40	-- (reference)	1420.0	-- (reference)
1 HSCs (*n* = 19)	6.69	0.29 (−0.08; 0.66)	1426.1	6.09 (−65.1; 77.3)
≥ 2 HSCs (*n* = 24)	6.87	0.47 (0.13; 0.82)**	1450.9	30.9 (−35.1; 96.9)

^a^Adjusted for age, sex, education, and intracranial volume

^b^Adjusted for age, sex, and education

^c^For hippocampal volume 39 measurements were available

**Significant difference in hippocampal volume between no HSCs present and ≥ 2 HSCs present with *p* < 0.01

In both study samples, no significant associations were observed between age, sex or vascular risk factors and presence of HSCs (Table 3). Unadjusted RR were similar to age and sex adjusted RR in both samples. The adjusted RR of having HSCs associated with age (per year increase) was 1.00 (95% CI: 0.98; 1.01) in the SMART-Medea sample and 1.01 (95% CI; 0.99; 1.03) in the PREDICT-MR sample. There was also no significant association between presence of HSCs and sex, educational level, and vascular risk factors.

**Table 3:** Results from the Poisson regression of the association of risk factors with presence of hippocampal cavities in the SMART-Medea and PREDICT-MR cohort

	SMART-Medea		PREDICT-MR	
	Adjusted for age and sex		Adjusted for age and sex	
	RR (95% CI)	P-value	RR (95% CI)	P-value
Age (1 per year increase)	1.00 (0.98; 1.01)^a^	0.62	1.01 (0.99; 1.03)^a^	0.18
Sex (women versus men)	1.16 (0.84; 1.60)^b^	0.38	0.94 (0.62; 1.43)^b^	0.77
Educational level (per level increase)	1.00 (0.93; 1.06)	0.91	0.99 (0.88; 1.11)	0.84
Cerebrovascular disease (yes vs. no)	1.27 (0.95; 1.70)	0.11	–	
Hypertension (yes vs. no)	1.05 (0.74; 1.49)	0.78	0.77 (0.51; 1.17)	0.22
Overweight (yes vs. no)	0.97 (0.69; 1.35)	0.84	0.94 (0.62; 1.42)	0.76
Hyperlipidemia (yes vs. no)	1.47 (0.87; 2.50)	0.15	0.89 (0.54; 1.48)	0.65
Diabetes Mellitus (yes vs. no)	0.78 (0.50; 1.21)	0.26	0.65 (0.32; 1.35)	0.25
Smoking (pack years)	1.00 (0.99; 1.01)	0.76	1.00 (0.98; 1.01)	0.18

^a^Adjusted for sex only

^b^Adjusted for age only

**Fig. 1:**
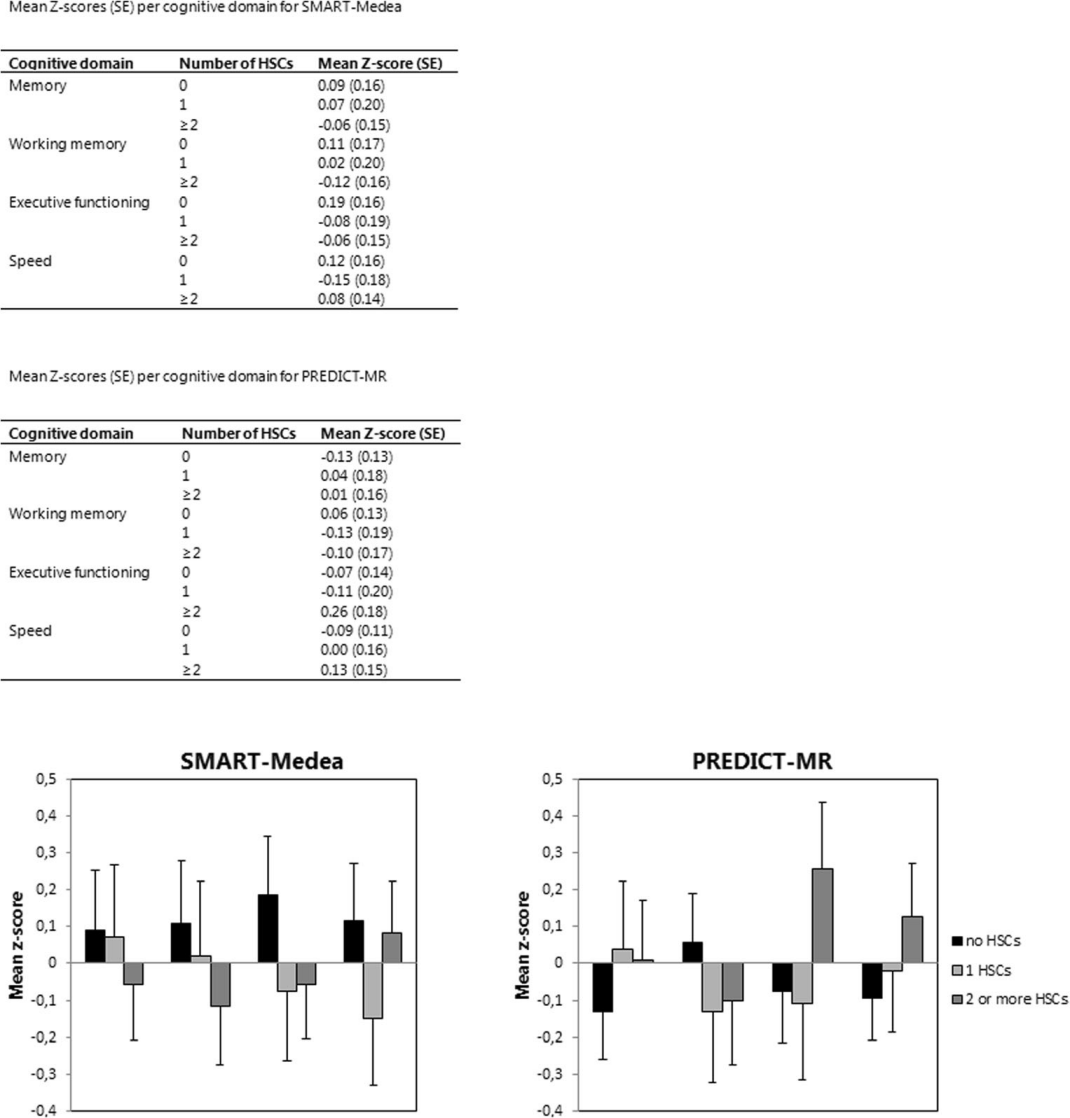
Mean Z-scores adjusted for age, sex and educational level per cognitive domain for both cohorts

Cognitive performance was not influenced by categories of number of HSCs in either study sample (Table 4). Figure 1 presents the Z-scores of all cognitive domains.

**Table 4:** Results from the linear regression analysis of the association of number of hippocampal cavities (0, 1 or 2 or more cavities) with Z-scores of cognitive performance

		Memory				Executive functioning			
		Unadjusted		Multivariable adjusted^a^		Unadjusted		Multivariable adjusted^a^	
		B (95% CI)	*P*-value	B (95% CI)	*P*-value	B (95% CI)	P-value	B (95% CI)	P-value
SMART-Medea	1 HSCs (n = 22)	0.07 (−0.48; 0.62)	0.80	−0.02 (−0.53; 0.49)	0.94	−0.17 (−0.71; 0.36)	0.52	−0.26 (−0.75; 0.23)	0.29
	≥2 HSCs (*n* = 37)	−0.15 (−0.62; 0.33)	0.58	−0.15 (−0.59; 0.30)	0.52	−0.30 (−0.77; 0.17)	0.21	−0.24 (−0.67; 0.19)	0.27
PREDICT-MR	1 HSCs (*n* = 20)	−0.03 (−0.58; 0.52)	0.92	0.17 (−0.27; 0.60)	0.45	−0.23 (−0.80; 0.35)	0.43	−0.04 (−0.53; 0.45)	0.88
	≥2 HSCs (n = 24)	−0.05 (−0.57; 0.47)	0.86	0.14 (−0.27; 0.55)	0.49	0.19 (−0.35; 0.74)	0.48	0.33 (−0.13; 0.79)	0.16
		Speed				Working memory			
		Unadjusted		Multivariable adjusted^a^		Unadjusted		Multivariable adjusted^a^	
		B (95% CI)	P-value	B (95% CI)	P-value	B (95% CI)	P-value	B (95% CI)	P-value
SMART-Medea	1 HSCs (n = 22)	−0.12 (−0.68; 0.44)	0.67	−0.26 (−0.74; 0.21)	0.27	−0.05 (−0.60; 0.51)	0.87	−0.09 (−0.61; 0.43)	0.74
	≥2 HSCs (n = 37)	−0.04 (−0.53; 0.45)	0.87	−0.03 (−0.45; 0.38)	0.87	−0.34 (−0.82; 0.14)	0.16	−0.23 (−0.68; 0.23)	0.33
PREDICT-MR	1 HSCs (n = 20)	−0.15 (−0.69; 0.39)	0.58	0.09 (−0.30; 0.49)	0.63	−0.29 (−0.76; 0.17)	0.22	−0.19 (−0.64; 0.26)	0.40
	≥2 HSCs (n = 24)	0.06 (−0.45; 0.57)	0.80	0.22 (−0.15; 0.59)	0.23	−0.20 (−0.65; 0.24)	0.37	−0.16 (−0.59; 0.27)	0.47

Reference category is no HSCs for all analyses (no HSCs for SMART-Medea *n* = 32; no HSCs for PREDICT-MR *n* = 40). B represents the mean difference in Z-score of cognitive performance for 1 vs. 0 HSC and for ≥2 vs. 0 HSCs

^a^Number of hippocampal cavities adjusted for age, sex, and education level

## Discussion

In this study, we observed that hippocampal sulcal cavities (HSCs) were very common on 1.5 T MRI. In patients with a history of vascular disease HSCs were observed in 65% while this was somewhat lower (52%) in the PREDICT-MR sample. In both study samples, no significant associations were observed between age, sex, vascular risk factors and the presence of HSCs, and no significant associations were observed between HSCs and cognitive functioning.

Previous studies with 1.5 T and 3 T MRI found similar frequencies of HSCs as we observed in the present study, with exception of our 7 T MRI study in which we found an occurrence of 97% of HSCs (Van Veluw et al. 2013). A review found a prevalence of 66% in patients with memory dysfunction and 47% in controls (Maller et al. 2011). Within other populations similar frequencies of HSCs were found (Maller et al. 2013); (Yao et al. 2014).

However, observed relationships of HSCs with risk factors and cognitive functioning are less consistent. We did not find a relationship between HSCs and age, which is consistent with other studies in healthy individuals and patients with Alzheimer’s disease (Van Veluw et al. 2013); (Li et al. 2006), but in contrast with other studies that included cognitively healthy elderly (Barboriak et al. 2000), adult volunteers of all ages (Yoneoka et al. 2002), or a mix of participants with normal cognition, mild cognitive impairment and AD (Chen et al. 2011); (Barboriak et al. 2000; Yoneoka et al. 2002); (Chen et al. 2011). It is possible that the relationship with age is weak and only visible when there is a large variation in age in the population studied. It is also possible that over the course of life HSCs become more visible due to atrophy of the brain tissue surrounding the cavity.

We also found no relationship with hypertension, which is in agreement with another study that included older persons without dementia (Barboriak et al. 2000)*.* In contrast, another study with community-dwelling elderly (Yao et al. 2014) did find that hypertension and both systolic and diastolic blood pressure were associated with an increased number of PVSs. Some other studies lacked information on vascular status of their control participants and patients with AD (Li et al. 2006), or included only healthy volunteers without hypertension (Yoneoka et al. 2002).

Our study did not find an association of the number of HSCs with cognitive functioning, which is in line with studies that included older persons from the community (Yao et al. 2014; Maclullich et al. 2004). However, a review showed that HSCs were more common in patients with memory impairment compared to controls (Maller et al. 2011). The increase in HSCs may thus result from diseases that underlie cognitive impairment (Maller et al. 2011), but they may also become more visible with age-related atrophy and not due to dementia itself (Heier et al. 1989).

Strengths of our study include the use of two different study samples. In this way, we were able to replicate our study in a second sample and thus increased the generalizability of our findings. Also, we did not use a size criterion and were therefore able to encompass the majority of HSCs irrespective of presumed etiology. Previous studies used different methods for researching the occurrence and effect of HSCs. Some rated HSCs assuming that they were perivascular spaces and therefore used a limitation in cavity size of 2 mm (Maclullich et al. 2004; Chen et al. 2011) or 3 mm (Yao et al. 2014; Zhang et al. 2014) in diameter. Larger spaces were determined as being focal lesions (Yao et al. 2014) or in some cases also as perivascular spaces when in an area of perforating arteries (Zhang et al. 2014).

A limitation of this study is the cross-sectional design, which does not allow any inferences about cause and consequence. Second, our participants were scanned with a 1.5 T MRI, which may underestimate the number of HSCs present, particularly HSCs that were smaller than 1 mm in diameter. In our previous study with use of 7 T MRI we observed HSCs in 97% of the participants. In that study, the sample size was smaller but most of these HSCs were presumed to be cavities filled with CSF, and 6% were thought to be of an ischemic subtype, perhaps microinfarcts as post-mortem pathology research on two HSCs revealed (Van Veluw et al. 2013). Other histology research suggested that the cavities were dPVS (Sasaki et al. 1993; Barboriak et al. 2000). Our current study lacked the possibility to do post-mortem verification of the cavities. However, the location of the HSCs and our observation that no associations exist with age, vascular risk factors, or cognitive functioning suggest that most of these cavities are remnants of the embryological development of the hippocampus (Kier et al. 1997; Hayman et al. 1998). Also, it is very likely that blood vessels are included within the sulcus during the fuse of the hippocampus walls as the arteries that feed the hippocampus penetrate there, among other places (Kier et al. 1997; Erdem et al. 1993), and thus these cavities may also be named PVS. Another limitation is that the FLAIR sequences at 1.5 T MRI did not allow to determine what proportion of HSCs may have been infarcts. Further, although we used two study samples, the size of each was relatively small and we cannot exclude the possibility that lack of power resulted in absence of statistically significant associations. However, when estimating the associations between age and common findings on MRI and cognitive functioning in the two study samples, we found significant associations with age in the expected direction (supplemental table 2). These findings imply that the power of this study is sufficient to find any positive associations. Finally, as we rated number of HSCs only, we do not know to what extent volume of cavities may have shown different associations.

In sum, in a population with a history of vascular disease and a population of primary care attendees not selected on disease status we found that hippocampal sulcal cavities are very common on 1.5 T MRI. However, no associations with age, sex, vascular risk factors or cognitive functioning were observed, suggesting that HSCs are mostly part of normal anatomic variation of the human hippocampus. To increase our understanding of the smaller proportion of cavities that might be pathological PVS or infarcts, we recommend future studies to include a large number of participants with a wide age range; and to incorporate ultra-high field MRI with a FLAIR sequence.

## Electronic supplementary material

ESM 1 (DOCX 17 kb)
