# Supplementary material for: Hippocampal sulcal cavities: prevalence, risk factors and association with cognitive performance. The SMART-Medea study and PREDICT-MR study
Source: Brain Imaging Behav. 2018 Jul 6;13(4):1093–102. doi: 10.1007/s11682-018-9916-y (PMC6647498; doi:10.1007/s11682-018-9916-y)
Supplement: Supplementary file 1 — (DOCX 17 kb) [file 11682_2018_9916_MOESM1_ESM.docx]

**Supplemental Table 1.** Neuropsychological tests, mean scores and standard deviation (S.D.)

| **Neuropsychological test** | **SMART-Medea**  **mean ± S.D.** | **PREDICT-MR**  **mean ± S.D.** |
| --- | --- | --- |
| MMSE | 28.6 ± 1.5 | 28.9 ± 1.1 |
| 15-WLT - total score (5 trials) | 37.9 ± 9.4 | 44.2 ± 9.8 |
| 15-WLT - delayed Recall | 7.6 ± 3.1 | 8.8 ± 3.5 |
| Rey Complex figure - delayed recall | 19.6 ± 6.3 | 17.6 ± 6.8 |
| Digit symbol substitution test (120 seconds) | 52.4 ± 15.7 | 63.0 ± 17.0 |
| Visual elevator (seconds per switch)^a^ | 5.2 ± 2.1 | 5.8 ± 8.9 |
| Brixton anticipation test^a^ (no. of errors) | 19.1 ± 6.4 | 18.4 ± 8.2 |
| Forward Digit Span –maximum span | 5.6 ± 1.1 | 5.7 ± 0.9 |
| Backward Digit Span – maximum span | 4.2 ± 1.2 | 4.3 ± 1.1 |
| Verbal Fluency – letter A (60 seconds) | 10.3 ± 4.0 | 11.0 ± 3.8 |
| Verbal Fluency – animals (120 seconds) | 30.2 ± 8.9 | 34.8 ± 9.0 |

^a^Neuropsychological tests in which higher scores represent poorer performance.

For all other tests higher score represent better performance.

**Supplemental Table 2.** Associations between age, and common findings on MRI, and associations between age and cognition in both study samples.

|  |  | **Hippocampal volume^1^** | **Total brain volume^1^** | **WMH volume^1^**  **(natural log-transformed)** | **Memory^2^** | **Executive functioning^2^** |
| --- | --- | --- | --- | --- | --- | --- |
|  |  | **B* (95% CI)** | **B (95% CI)** | **B (95% CI)** | **B (95% CI)** | **B (95% CI)** |
| SMART-Medea | age | -0.02  (-0.03; -0.01) | -2.76  (-3.55; -1.97) | 0.06  (0.04; 0.08) | -0.04  (-0.06; -0.02) | -0.04  (-0.06; -0.02) |
| PREDICT-MR | age | -0.01  (-0.02; 0.00) | -3.41  (-4.52; -2.30) | 0.08  (0.04; 0.12) | -0.05  (-0.06; -0.03) | -0.04  (-0.06; -0.02) |

^1^Analyses with age and common finding on MRI were adjusted for sex and intracranial volume.

^2^Analyses with age and cognition were adjusted for sex and education.

*B is the unstandardized regression coefficient.

WMH=white matter hyperintensity
